# Neonatal Respiratory Outcomes in Placenta Previa with Histopathologically Confirmed Placenta Accreta Spectrum

**DOI:** 10.3390/jcm15155891

**Published:** 2026-07-28

**Authors:** Marta Mlodawska, Anna Zmelonek-Znamirowska, Grzegorz Swiercz, Jay Mehta, Jakub Mlodawski

**Affiliations:** 1 Collegium Medicum, Jan Kochanowski University in Kielce, Zeromskiego Street 5, 25-369 Kielce, Poland; grzegorz.swiercz@ujk.edu.pl (G.S.); jakub.mlodawski@ujk.edu.pl (J.M.); 2 Neonatology Clinic, Provincial Combined Hospital in Kielce, Grunwaldzka 45, 25-736 Kielce, Poland; 3 Clinic of Obstetrics and Gynecology, Provincial Combined Hospital in Kielce, Grunwaldzka 45, 25-736 Kielce, Poland; 4 Shree IVF and Endometriosis Hospital, Mumbai 400077, Maharashtra, India

**Keywords:** placenta accreta spectrum, placenta praevia, neonatal outcomes, respiratory support, resuscitation at birth, supplemental oxygen

## Abstract

**Background and Objectives**: Placenta accreta spectrum (PAS) complicating placenta praevia is associated with increased maternal morbidity; however, neonatal respiratory burden may be underappreciated. We compared neonatal outcomes between pregnancies complicated by placenta praevia with and without histopathologically confirmed PAS, with additional adjustment of respiratory outcomes for gestational age at delivery. **Materials and Methods**: We conducted a retrospective single-centre cohort study (*n* = 82). Based on placental histopathology, women were classified as PAS (*n* = 37; accreta/increta/percreta) or non-PAS (*n* = 45). Respiratory endpoints included respiratory resuscitation at birth, non-invasive respiratory support, and supplemental oxygen requirement with fraction of inspired oxygen (FiO_2_) > 0.21 during neonatal intensive care unit (NICU) hospitalization. Logistic regression models adjusted respiratory endpoints for gestational age. **Results**: Data were available for 80/82 neonates. Compared with non-PAS, PAS cases had higher rates of respiratory resuscitation at birth (26/36 [72.2%] vs. 19/44 [43.2%], *p* = 0.013), non-invasive respiratory support (31/36 [86.1%] vs. 24/44 [54.5%], *p* = 0.003), and supplemental oxygen requirement (29/36 [80.6%] vs. 21/44 [47.7%], *p* = 0.003). After adjustment for gestational age, PAS remained associated with respiratory resuscitation (adjusted odds ratio [aOR] 3.34, 95% confidence interval [CI] 1.21–9.27; *p* = 0.020) and supplemental oxygen requirement (aOR 4.60, 95% CI 1.35–15.75; *p* = 0.015). The association with non-invasive respiratory support did not reach statistical significance (aOR 4.46, 95% CI 0.92–21.68; *p* = 0.064). **Conclusions**: Histopathologically confirmed PAS was associated with higher neonatal respiratory support requirements after adjustment for gestational age, supporting anticipatory obstetric–neonatal planning when PAS is suspected.

## 1. Introduction

Placenta accreta spectrum (PAS), previously termed morbidly adherent placenta, is a broad term describing abnormal placental adherence or invasion into the myometrium and sometimes into, or beyond, the uterine serosa. This abnormal trophoblastic invasion of the uterine wall prevents spontaneous placental separation after delivery and increases the risk of life-threatening haemorrhage, usually requiring peripartum hysterectomy. PAS encompasses three subtypes: (1) placenta accreta (or creta), in which chorionic villi attach to the decidua or superficially to the myometrium without deep invasion (63%); (2) placenta increta, in which villi invade the myometrium (15%); and (3) placenta percreta, in which villi penetrate through the myometrium to the uterine serosa or adjacent organs (22%) [1].

The incidence of PAS is estimated at 0.17% (range 0.01–1.1%) [1]. This incidence has increased in recent decades, mainly due to the rising rate of caesarean delivery [2].

In most cases, PAS is thought to result from placental implantation in an area of defective decidualisation caused by pre-existing disruption of the endometrial–myometrial interface. The most important risk factor is placenta praevia in a woman with a previous caesarean section. Therefore, when placenta praevia is diagnosed, the possibility of PAS should always be considered. In the presence of placenta praevia, the risk of PAS increases with the number of prior caesarean sections: first (3%), second (11%), third (40%), fourth (61%), fifth (67%) [3]. In the absence of placenta praevia, the incidence of PAS among women undergoing caesarean section is much lower: first (0.03%), second (0.2%), third (0.1%), fourth (0.8%), and fifth (4.7%) [3]. Placenta praevia is an independent risk factor for PAS (odds ratio [OR] 54, 95% confidence interval [CI] 18–166) [4]. In cases of PAS without concomitant placenta praevia, the risk of maternal complications is lower, probably due to a reduced likelihood of deep invasion or extensive adherence [5].

Other risk factors include manual removal of the placenta, maternal age over 35 years, and previous surgery involving the myometrium [6].

PAS can be suspected prenatally on ultrasound examination. Clinical manifestations during pregnancy may include vaginal bleeding (most often due to coexisting placenta praevia), pelvic pain, preterm birth, and preterm prelabour rupture of membranes [7]. Unrecognised PAS may present as life-threatening postpartum haemorrhage during attempted manual placental removal, resulting from failure of separation of a placenta firmly attached to the uterine wall.

Neonatal respiratory morbidity is a clinically relevant endpoint in pregnancies complicated by placenta praevia and PAS. These pregnancies are frequently delivered by caesarean section before the onset of labour and often before term in order to reduce the risk of emergency haemorrhage and uncontrolled placental disruption. Lower gestational age at birth, absence of labour before caesarean delivery, maternal haemorrhage, operative complexity, and the need for general anaesthesia may all influence early neonatal respiratory adaptation and increase the need for oxygen supplementation, respiratory support, or neonatal intensive care unit admission [8,9,10].

Although maternal morbidity associated with PAS has been widely described, neonatal outcomes, particularly respiratory outcomes, remain less well characterised. Available studies suggest that PAS may be associated with increased rates of adverse neonatal outcomes, including neonatal intensive care unit (NICU) admission and respiratory support; however, it remains uncertain to what extent these outcomes are attributable to invasive placentation itself, coexisting placenta praevia, lower gestational age at delivery, anaesthetic management, maternal haemorrhage, or the complexity of peripartum surgical care [9,10].

Therefore, the aim of our study was to compare neonatal respiratory outcomes in pregnancies complicated by placenta praevia alone versus placenta praevia with placenta accreta spectrum.

## 2. Materials and Methods

A retrospective cohort study was conducted at the Department of Obstetrics and Gynecology of the Provincial Combined Hospital in Kielce. The medical records of all consecutive patients with placenta praevia who delivered at our institution between 2014 and 2021 were screened. The study was approved by the Bioethics Committee of Jan Kochanowski University in Kielce (resolution no. 23/2022). The study was reported in accordance with the Strengthening the Reporting of Observational Studies in Epidemiology (STROBE) recommendations. The completed STROBE checklist for cohort studies is provided as Supplementary File S1.

**Figure 1:**
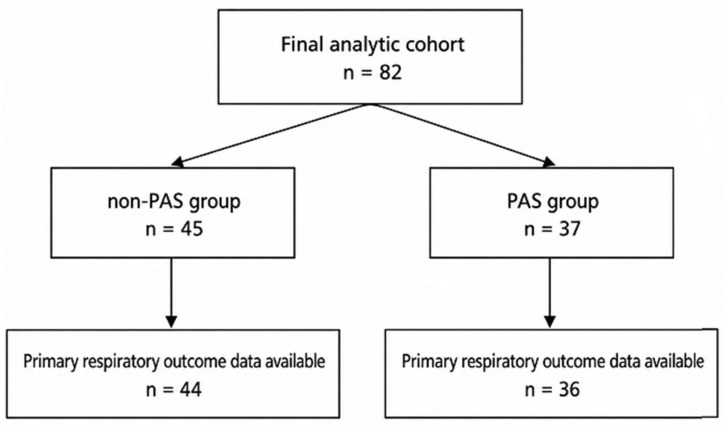
Flow diagram of the final analytic cohort, histopathological classification into PAS and non-PAS groups, and availability of data for the primary respiratory outcomes. PAS, placenta accreta spectrum.

The inclusion criteria were placenta praevia, singleton pregnancy, delivery by caesarean section at our institution, and available placental histopathological assessment. Cases without placental histopathological assessment were excluded. Based on histopathological examination, patients were classified as PAS or non-PAS. In all PAS cases, the diagnosis was established on hysterectomy specimens. The PAS group comprised cases of placenta accreta, increta, or percreta, whereas the non-PAS group showed no histopathological evidence of placental invasion. The final analytic cohort, histopathological classification into PAS and non-PAS groups, and availability of data for the primary respiratory outcomes are presented in Figure 1. The numbers of patients initially screened and excluded could not be reliably reconstructed from the available retrospective records.

Pregnancies in which PAS was suspected prenatally were managed using multidisciplinary perinatal planning. In clinically stable patients, caesarean delivery was typically scheduled at approximately 34 weeks of gestation, according to the institutional management protocol applicable during the study period. Emergency caesarean delivery was performed in cases of active antepartum bleeding, onset of labour, maternal or fetal compromise, or other urgent clinical indications. Antenatal corticosteroids were administered according to gestational age and the anticipated timing of delivery, in accordance with the local clinical protocol applicable during the study period.

Maternal and obstetric data collected from medical records included maternal age, parity, gestational age at delivery, urgency of caesarean delivery (elective vs. emergency), and antenatal corticosteroid administration. Neonatal data were extracted from neonatal medical records and included birth weight, neonatal sex, Apgar scores at 1, 5, and 10 min, umbilical cord blood pH, and the need for respiratory or cardiorespiratory resuscitation at birth. Data regarding non-invasive respiratory support, mechanical ventilation, supplemental oxygen with fraction of inspired oxygen (FiO_2_) > 0.21 during neonatal intensive care unit (NICU) hospitalization, and surfactant administration were also collected. Neonatal complications assessed included respiratory distress syndrome (RDS), transient tachypnoea of the newborn (TTN), congenital pneumonia, intraventricular haemorrhage (IVH), congenital infection, secondary infection, anaemia, and anaemia requiring red blood cell transfusion. Missing data for individual neonatal outcomes were reflected in the denominators reported in the Results and tables. Missing observations resulted from incomplete documentation or the absence of the relevant variable in the available retrospective medical records. No missing data were imputed. Descriptive and univariable analyses were performed using the available observations for each outcome, whereas multivariable sensitivity analyses were conducted using complete-case data.

Respiratory resuscitation at birth was defined as any positive-pressure respiratory support delivered using a Neopuff device, including sustained inflation breaths, positive end-expiratory pressure (PEEP), or peak inspiratory pressure/PEEP ventilation, depending on the clinical condition of the newborn. Passive oxygen supplementation alone, without positive-pressure respiratory support, was not classified as respiratory resuscitation. Cardiorespiratory resuscitation was defined as chest compressions, with or without the administration of resuscitation medications, in addition to respiratory support.

Non-invasive respiratory support during NICU hospitalization was defined as continuous positive airway pressure (CPAP) or non-invasive ventilation (NIV) with positive inspiratory pressure. High-flow nasal cannula was not used in this cohort. Mechanical ventilation was defined as invasive ventilation through an endotracheal tube. Oxygen requirement was defined as the need for supplemental oxygen with FiO_2_ > 0.21 during NICU hospitalization. Surfactant administration was recorded when exogenous surfactant was administered during NICU care.

RDS, TTN, congenital pneumonia, IVH, congenital infection, secondary infection, anaemia, and red blood cell transfusion were recorded based on neonatal medical records and discharge diagnoses. Congenital infection was additionally evaluated in relation to available inflammatory markers, including C-reactive protein (CRP), procalcitonin, and blood culture results. Anaemia was defined as a haemoglobin concentration below the age-specific reference range. The need for red blood cell transfusion was determined according to the restrictive transfusion criteria used in the neonatal unit during the study period.

Statistical analyses were performed using Python version 3.11.2 (Python Software Foundation, Beaverton, OR, USA) with the SciPy and statsmodels libraries. Continuous variables are presented as medians with interquartile ranges (IQRs), and between-group comparisons were performed using the Mann–Whitney U test. Categorical variables are presented as counts and percentages and were compared using the χ^2^ test or Fisher’s exact test, as appropriate. For categorical outcomes, odds ratios (ORs) with 95% confidence intervals (CIs) were calculated. The Haldane–Anscombe correction was applied when zero cells were present in contingency tables.

To evaluate the independent association between PAS and neonatal respiratory outcomes after accounting for gestational age at delivery, logistic regression analyses were performed for three primary respiratory endpoints: (1) respiratory resuscitation at birth using Neopuff-assisted support, (2) non-invasive respiratory support during NICU hospitalization, and (3) supplemental oxygen requirement with FiO_2_ > 0.21 during NICU hospitalization. PAS status and gestational age at delivery were included as independent variables in the primary models.

To reduce the risk of overfitting, the primary logistic regression models were intentionally parsimonious. Covariates were selected based on their clinical relevance to neonatal respiratory outcomes, the study objective, data availability, and events-per-variable (EPV) constraints. The effective EPV ratio was assessed using the smaller of the event and non-event counts for each endpoint. The effective EPV ratio was above 10 for all three primary respiratory endpoints. Extended models additionally adjusted for urgency of caesarean delivery and antenatal corticosteroid administration. These models were treated as exploratory sensitivity analyses because of the lower events-per-variable ratio and limited sample size and were performed using complete-case data (*n* = 80). No formal correction for multiple comparisons was applied; analyses of secondary neonatal outcomes were considered exploratory.

Because all included pregnancies were singleton pregnancies delivered by caesarean section, plurality and mode of delivery were not entered as covariates. Neonatal sex was not included in the primary multivariable models because of the limited sample size and events-per-variable constraints.

Logistic regression results are presented as adjusted odds ratios (aORs) with 95% CIs. All statistical tests were two-sided, and a *p* value < 0.05 was considered statistically significant.

No prospective sample size calculation was performed because of the retrospective design of the study and the rarity of PAS among patients with placenta praevia. All eligible cases meeting the inclusion criteria during the study period were included.

## 3. Results

A total of 82 women with placenta praevia who delivered between 2014 and 2021 were included; 37 were classified as PAS (accreta, increta, or percreta) and 45 as non-PAS based on placental histopathology. Baseline demographic and obstetric characteristics are shown in Table 1.

Maternal age was similar between groups: 34 (30–36) vs. 35 (32–38) years (*p* = 0.100). Gestational age at delivery was also comparable: 35 (32–37) vs. 34 (33–36) weeks (*p* = 0.253). Birth weight, available for 76/82 neonates, tended to be lower in the PAS group: 2245 (2055–2513) vs. 2595 (1993–3123) g (*p* = 0.061). Neonatal sex, available for 76/82 neonates, did not differ (male: 23/42 [54.8%] vs. 18/34 [52.9%], *p* = 1.000). Multiparity (≥2 deliveries; available for 81/82 women) was more frequent in the PAS group (36/37 [97.3%] vs. 35/44 [79.5%], *p* = 0.018).

Emergency caesarean delivery (available for 81/82 women) was less frequent in the PAS group (7/37 [18.9%] vs. 26/44 [59.1%], *p* < 0.001). Rates of antenatal corticosteroid administration did not differ between groups.(available for 80/82 women) (17/36 [47.2%] vs. 17/44 [38.6%], *p* = 0.499).

Neonatal outcomes are summarized in Table 2, with denominators varying due to missing neonatal chart data for selected variables. Umbilical cord blood pH was available in 53/82 neonates (non-PAS 35/45; PAS 18/37) and no case had pH < 7.2. Low Apgar scores were uncommon: Apgar < 7 at 1 min occurred in 9/43 vs. 9/34 (*p* = 0.598), at 5 min in 2/43 vs. 6/34 (*p* = 0.129), and at 10 min in 1/43 vs. 1/34 (*p* = 1.000) for non-PAS vs. PAS, respectively.

Respiratory endpoints were available for 80/82 neonates (non-PAS: 44/45; PAS: 36/37). Compared with neonates in the non-PAS group, those in the PAS group more frequently required respiratory resuscitation at birth (26/36 [72.2%] vs. 19/44 [43.2%]; OR 3.42, 95% CI 1.33–8.78; *p* = 0.013), non-invasive respiratory support during NICU hospitalization (31/36 [86.1%] vs. 24/44 [54.5%]; OR 5.17, 95% CI 1.69–15.76; *p* = 0.003), and supplemental oxygen with FiO_2_ > 0.21 during NICU hospitalization (29/36 [80.6%] vs. 21/44 [47.7%]; OR 4.54, 95% CI 1.64–12.53; *p* = 0.003). Cardiorespiratory resuscitation at birth was infrequent and did not differ between groups (4/36 [11.1%] vs. 4/44 [9.1%], *p* = 1.000).

Mechanical ventilation and RDS were numerically more frequent in the PAS group, although these differences did not reach statistical significance (mechanical ventilation: 8/36 [22.2%] vs. 4/44 [9.1%], *p* = 0.124; RDS: 8/29 [27.6%] vs. 5/32 [15.6%], *p* = 0.351). Surfactant administration was also numerically more common in the PAS group, but without statistical significance (12/36 [33.3%] vs. 9/44 [20.5%], *p* = 0.212). Data for RDS and IVH were incomplete, being available for 61/82 and 66/82 neonates, respectively; IVH rates were similar between groups (4/32 [12.5%] vs. 3/34 [8.8%], *p* = 0.705). Given the limited sample size and incomplete data, these secondary outcomes should be interpreted cautiously, and the lack of statistical significance should not be taken as evidence of no difference between groups.

No statistically significant between-group differences were observed in infectious or haematological outcomes within the available data. Congenital infection occurred in 12/34 neonates in the PAS group and 10/44 neonates in the non-PAS group (*p* = 0.311), secondary infection occurred in 4/34 and 1/40 neonates, respectively (*p* = 0.173), anaemia occurred in 17/37 and 18/44 neonates, respectively (*p* = 0.660), and red blood cell transfusion was required in 16/36 and 16/44 neonates, respectively (*p* = 0.499).

Logistic regression analysis adjusted for gestational age at delivery (Table 3) showed higher odds of respiratory resuscitation at birth in the PAS group (aOR 3.34, 95% CI 1.21–9.27; *p* = 0.020) and a higher supplemental oxygen requirement with FiO_2_ > 0.21 during NICU hospitalization (aOR 4.60, 95% CI 1.35–15.75; *p* = 0.015). The association between PAS and non-invasive respiratory support during NICU hospitalization did not reach statistical significance (aOR 4.46, 95% CI 0.92–21.68; *p* = 0.064).

In complete-case sensitivity analyses (n = 80) additionally adjusted for urgency of caesarean delivery, effect estimates for PAS remained similar for respiratory resuscitation at birth (aOR 3.27, 95% CI 1.06–10.04), supplemental oxygen requirement with FiO_2_ > 0.21 during NICU hospitalization (aOR 4.44, 95% CI 1.20–16.35), and non-invasive respiratory support during NICU hospitalization (aOR 4.67, 95% CI 0.91–23.85). Emergency caesarean delivery was not independently associated with any of the three respiratory endpoints (all *p* ≥ 0.809). In sensitivity models additionally adjusted for antenatal corticosteroid administration, the effect estimates for PAS remained similar. PAS remained associated with respiratory resuscitation at birth (aOR 3.50, 95% CI 1.11–11.01; *p* = 0.032) and supplemental oxygen requirement with FiO_2_ > 0.21 during NICU hospitalization (aOR 4.37, 95% CI 1.16–16.52; *p* = 0.030), whereas its association with non-invasive respiratory support remained non-significant (aOR 4.65, 95% CI 0.91–23.78; *p* = 0.065). Antenatal corticosteroid administration was not independently associated with respiratory resuscitation at birth (aOR 0.59, 95% CI 0.20–1.75; *p* = 0.344), non-invasive respiratory support (aOR 1.07, 95% CI 0.21–5.59; *p* = 0.935), or supplemental oxygen requirement with FiO_2_ > 0.21 during NICU hospitalization (aOR 2.62, 95% CI 0.70–9.73; *p* = 0.151). Higher gestational age at delivery was independently associated with lower odds of all three respiratory endpoints across the models.

## 4. Discussion

In cases of placenta praevia, the presence of sonographic features suggesting placental invasion obliges obstetricians to deliver earlier in order to reduce the risk of severe maternal complications, such as peripartum hysterectomy due to massive haemorrhage and failure of uterine contraction when the placental tissue infiltrates the uterine wall and cannot be separated. Because of the increased risk of massive intrapartum haemorrhage, the markedly elevated maternal morbidity and potential mortality, these procedures are performed within a multidisciplinary team, often including a urologist and/or consulting gynaecologic oncologist, with several units of blood cross-matched and immediately available for possible transfusion. In our department, such elective caesarean sections are scheduled at approximately 34 weeks’ gestation, in line with current recommendations [8].

In our cohort, multiparity was more frequent in the PAS group than in the non-PAS group (97.3% vs. 79.5%; *p* = 0.018), which is consistent with previous reports identifying increasing parity as a risk factor for PAS [2]. Emergency caesarean delivery occurred less frequently in the PAS group than in the non-PAS group (18.9% vs. 59.1%). This difference may reflect prenatal recognition of PAS in most affected cases and our unit’s practice of scheduling caesarean delivery at approximately 34 weeks of gestation. In our clinical experience, antepartum bleeding is the most frequent indication for emergency caesarean delivery in pregnancies complicated by placenta praevia.

Despite the difference in the frequency of emergency caesarean delivery, median gestational age at delivery and median birthweight did not differ significantly between the groups. This suggests that PAS status was not the only determinant of the timing of delivery in this cohort. The substantial burden of preterm delivery in both groups may also be related to placenta praevia and associated antepartum bleeding. This interpretation is consistent with a 2015 meta-analysis showing that placenta praevia was associated with a three- to fivefold higher risk of preterm birth (preterm birth rate 44%; RR 5.32, 95% CI 4.39–6.45) [11].

The literature includes studies comparing neonatal outcomes of mothers with PAS with those of selected control groups not limited to patients with placenta praevia. These studies have demonstrated an increased risk of neonatal complications, including preterm delivery (10.7% vs. 1%, *p* < 0.001; OR 12.1, 95% CI 3.7–39.9) and small-for-gestational-age infants (27.3% vs. 14%, *p* < 0.001; OR 5.05, 95% CI 1.46–3.28) [12]. A strength of our study is that PAS cases were compared specifically with a control group of patients with placenta praevia without PAS, with no significant differences in gestational age at delivery or birth weight. In contrast, we observed a higher respiratory burden in the PAS group, including more frequent respiratory resuscitation at birth, greater need for non-invasive respiratory support during NICU hospitalization, and more frequent supplemental oxygen requirement. After adjustment for gestational age, PAS remained associated with respiratory resuscitation at birth and oxygen requirement, whereas the association with non-invasive respiratory support did not reach statistical significance. These findings suggest that, in this cohort, PAS was associated with increased neonatal respiratory support requirements beyond the effect of gestational age alone, although this observation requires confirmation in larger prospective studies.

These observations may have practical implications for perinatal planning. When PAS is suspected prenatally, the neonatal team should be informed in advance and present at delivery, with readiness to provide positive-pressure respiratory support immediately after birth and non-invasive respiratory support during NICU care. From an obstetric perspective, planned late-preterm delivery in PAS represents a balance between reducing the risk of uncontrolled maternal haemorrhage and accepting the neonatal respiratory burden associated with lower gestational age. Therefore, within the recommended strategy of planned late-preterm delivery for PAS, our findings emphasise the importance of coordinated obstetric–neonatal preparation, including timely neonatal team involvement and appropriate antenatal corticosteroid administration according to gestational age and the expected timing of delivery.

A possible, but purely hypothetical, explanation for the observed association may involve impaired early neonatal pulmonary vascular adaptation. Failure of the physiological decline in pulmonary vascular resistance after birth may result in persistence of a transitional circulation pattern, reduced pulmonary blood flow, impaired pulmonary oxygen uptake, and clinical respiratory failure. Previous studies have suggested that vascular endothelial growth factor (VEGF) plays an important role in normal fetal pulmonary vascular development and neonatal pulmonary circulation [13]. However, our study did not include biomarker, molecular, or imaging data that would allow this mechanism to be tested directly. Therefore, the proposed role of abnormal placental vascular remodelling, including potential VEGF-related pathways, should be interpreted only as a speculative biological hypothesis. Further studies are needed to investigate this potential mechanism.

The incidence of congenital infection did not differ between groups. However, this secondary outcome should be interpreted cautiously, because most cases classified as congenital infection were based on isolated procalcitonin elevation without concomitant CRP increase or positive blood cultures. Since procalcitonin may be elevated in the immediate neonatal period also in non-infectious conditions, including respiratory distress or perinatal stress [14,15], congenital infection findings should be interpreted cautiously.

Several limitations should be acknowledged. This was a single-centre retrospective study, and no prospective sample size calculation was performed. Selection bias related to the requirement for histopathological assessment cannot be excluded, and the single-centre setting may limit generalisability to institutions with different obstetric and neonatal care practices. Although all eligible cases from the study period were included, the rarity of PAS limited the final sample size. Consequently, the study may have been insufficiently powered to detect differences in less frequent secondary outcomes, including mechanical ventilation and RDS, for which the observed rates were numerically higher in the PAS group but did not reach statistical significance. These findings should not be interpreted as evidence of no difference between the groups. Missing data for selected neonatal outcomes may have further reduced statistical power and introduced bias if data availability was related to neonatal condition. Another limitation is the relatively low events-per-variable ratio in the extended sensitivity models. The primary logistic regression models were intentionally kept parsimonious and included only PAS status and gestational age, whereas models additionally adjusted for emergency caesarean delivery and antenatal corticosteroid administration had lower EPV and should therefore be regarded as exploratory sensitivity analyses rather than fully confirmatory multivariable models. Formal assessments of multicollinearity, model calibration, and goodness-of-fit were not performed, and no correction for multiple comparisons was applied. Residual confounding by additional maternal and obstetric factors cannot be excluded, including parity, the number of previous caesarean deliveries, maternal comorbidities, the frequency or severity of antepartum bleeding, estimated maternal blood loss, transfusion volume, anaesthesia method, haemodynamic instability, and the detailed intraoperative course. We did not evaluate the impact of the depth of placental invasion on neonatal outcomes; PAS was analysed as a single histopathologically confirmed category without separate assessment of accreta, increta, and percreta. Previous research found no association between the depth of placental invasion and neonatal outcomes [16]. Furthermore, non-invasive respiratory support was analysed as a binary clinical endpoint encompassing CPAP or NIV with positive inspiratory pressure. CPAP and NIV were not analysed separately, and detailed pressure settings, escalation pathways, and mode-specific severity were not assessed. Larger multicentre cohorts are needed to confirm these findings and better characterise the severity and trajectory of neonatal respiratory morbidity in pregnancies complicated by PAS.

## 5. Conclusions

1. Among pregnancies complicated by placenta praevia, gestational age at delivery and neonatal birth weight were similar between the PAS and non-PAS groups.
2. PAS was associated with higher neonatal respiratory support requirements. The associations with respiratory resuscitation at birth and supplemental oxygen requirement persisted after adjustment for gestational age at delivery and remained similar in exploratory sensitivity analyses. However, residual confounding and differences in obstetric management or delivery pathways may have contributed to these findings, and causality cannot be established.
3. Emergency caesarean delivery was less frequent in the PAS group, which may reflect prenatal recognition and planned delivery management.
4. Higher gestational age at delivery was independently associated with lower odds of respiratory interventions across the regression models (aOR range per additional week: 0.30–0.74; all *p* ≤ 0.001).

## Figures and Tables

**Table 1:** Baseline maternal and obstetric characteristics.

Characteristic	Non-PAS (n = 45)	PAS (n = 37)	*p*
Maternal age, years	34 (30–36)	35 (32–38)	0.100
Gestational age at delivery, weeks	35 (32–37)	34 (33–36)	0.253
Birth weight, g	2595 (1993–3123)	2245 (2055–2513)	0.061
Male sex	23/42 (54.8%)	18/34 (52.9%)	1.000
Multiparous (≥2 deliveries)	35/44 (79.5%)	36/37 (97.3%)	0.018
Antenatal corticosteroids	17/44 (38.6%)	17/36 (47.2%)	0.499
Emergency caesarean delivery	26/44 (59.1%)	7/37 (18.9%)	<0.001

Data are presented as median (Q1–Q3) or *n/N* (%), where N denotes the number of available observations. PAS, placenta accreta spectrum; Q1, first quartile; Q3, third quartile. *p* values were calculated using Fisher’s exact test for categorical variables and the Mann–Whitney U test for continuous variables.

**Table 2:** Neonatal outcomes. Data are presented as *n*/*N* (%), where *N* denotes the number of available observations.

Outcome	Non-PAS (n = 45)	PAS (n = 37)	*p*
Umbilical cord pH < 7.2	0/35 (0.0%)	0/18 (0.0%)	1.000
Apgar 1′ < 4	1/43 (2.3%)	0/34 (0.0%)	1.000
Apgar 1′ < 7	9/43 (20.9%)	9/34 (26.5%)	0.598
Apgar 5′ < 4	0/43 (0.0%)	1/34 (2.9%)	0.442
Apgar 5′ < 7	2/43 (4.7%)	6/34 (17.6%)	0.129
Apgar 10′ < 4	0/43 (0.0%)	0/34 (0.0%)	1.000
Apgar 10′ < 7	1/43 (2.3%)	1/34 (2.9%)	1.000
Respiratory resuscitation at birth	19/44 (43.2%)	26/36 (72.2%)	0.013
Cardiorespiratory resuscitation at birth	4/44 (9.1%)	4/36 (11.1%)	1.000
Congenital infection	10/44 (22.7%)	12/34 (35.3%)	0.311
Isolated increase in procalcitonin value	8/44 (18.2%)	10/34 (29.4%)	0.286
Secondary infection	1/40 (2.5%)	4/34 (11.8%)	0.173
Anaemia	18/44 (40.9%)	17/37 (45.9%)	0.660
Red blood cell transfusion	16/44 (36.4%)	16/36 (44.4%)	0.499
Congenital pneumonia	6/44 (13.6%)	2/36 (5.6%)	0.284
Transient tachypnoea of the newborn (TTN)	1/44 (2.3%)	2/36 (5.6%)	0.585
Respiratory distress syndrome (RDS), any grade	5/32 (15.6%)	8/29 (27.6%)	0.351
Surfactant administration	9/44 (20.5%)	12/36 (33.3%)	0.212
Intraventricular haemorrhage (IVH), any grade	3/34 (8.8%)	4/32 (12.5%)	0.705
FiO_2_ > 0.5 during NICU hospitalization	3/44 (6.8%)	6/36 (16.7%)	0.286
FiO_2_ > 0.4 during NICU hospitalization	9/44 (20.5%)	14/36 (38.9%)	0.086
FiO_2_ > 0.3 during NICU hospitalization	13/44 (29.5%)	19/36 (52.8%)	0.042
FiO_2_ > 0.21 during NICU hospitalization	21/44 (47.7%)	29/36 (80.6%)	0.003
Non-invasive respiratory support	24/44 (54.5%)	31/36 (86.1%)	0.003
Mechanical ventilation	4/44 (9.1%)	8/36 (22.2%)	0.124

PAS, placenta accreta spectrum; FiO_2_, fraction of inspired oxygen; NICU, neonatal intensive care unit.

**Table 3:** Logistic regression analysis of the association between PAS and selected neonatal respiratory outcomes. Primary models were adjusted for gestational age at delivery; sensitivity models were additionally adjusted for urgency of caesarean delivery and antenatal corticosteroid administration.

Dependent Variable	Independent Variable	aOR	95% CI	*p*
Respiratory resuscitation at birth	PAS (vs. non-PAS)	3.34	1.21–9.27	0.020
Respiratory resuscitation at birth	Gestational age (per week)	0.74	0.62–0.89	0.001
Sensitivity model: additionally adjusted for urgency of caesarean delivery				
Respiratory resuscitation at birth	PAS (vs. non-PAS)	3.27	1.06–10.04	0.039
Respiratory resuscitation at birth	Gestational age (per week)	0.74	0.61–0.90	0.003
Respiratory resuscitation at birth	Emergency caesarean delivery (vs. elective)	0.94	0.28–3.16	0.924
Sensitivity model: additionally adjusted for urgency of caesarean delivery and antenatal corticosteroid administration				
Respiratory resuscitation at birth	PAS (vs. non-PAS)	3.50	1.11–11.01	0.032
Respiratory resuscitation at birth	Gestational age (per week)	0.73	0.60–0.89	0.002
Respiratory resuscitation at birth	Emergency caesarean delivery (vs. elective)	1.02	0.30–3.50	0.970
Respiratory resuscitation at birth	Antenatal corticosteroid administration	0.59	0.20–1.75	0.344
Non-invasive respiratory support	PAS (vs. non-PAS)	4.46	0.92–21.68	0.064
Non-invasive respiratory support	Gestational age (per week)	0.30	0.16–0.56	<0.001
Sensitivity model: additionally adjusted for urgency of caesarean delivery				
Non-invasive respiratory support	PAS (vs. non-PAS)	4.67	0.91–23.85	0.064
Non-invasive respiratory support	Gestational age (per week)	0.30	0.16–0.57	<0.001
Non-invasive respiratory support	Emergency caesarean delivery (vs. elective)	1.23	0.23–6.68	0.809
Sensitivity model: additionally adjusted for urgency of caesarean delivery and antenatal corticosteroid administration				
Non-invasive respiratory support	PAS (vs. non-PAS)	4.65	0.91–23.78	0.065
Non-invasive respiratory support	Gestational age (per week)	0.30	0.16–0.58	<0.001
Non-invasive respiratory support	Emergency caesarean delivery (vs. elective)	1.22	0.22–6.80	0.825
Non-invasive respiratory support	Antenatal corticosteroid administration	1.07	0.21–5.59	0.935
FiO_2_ > 0.21 in NICU	PAS (vs. non-PAS)	4.60	1.35–15.75	0.015
FiO_2_ > 0.21 in NICU	Gestational age (per week)	0.54	0.40–0.74	<0.001
Sensitivity model: additionally adjusted for urgency of caesarean delivery				
FiO_2_ > 0.21 in NICU	PAS (vs. non-PAS)	4.44	1.20–16.35	0.025
FiO_2_ > 0.21 in NICU	Gestational age (per week)	0.54	0.39–0.74	<0.001
FiO_2_ > 0.21 in NICU	Emergency caesarean delivery (vs. elective)	0.89	0.23–3.53	0.870
Sensitivity model: additionally adjusted for urgency of caesarean delivery and antenatal corticosteroid administration				
FiO_2_ > 0.21 in NICU	PAS (vs. non-PAS)	4.37	1.16–16.52	0.030
FiO_2_ > 0.21 in NICU	Gestational age (per week)	0.54	0.39–0.75	<0.001
FiO_2_ > 0.21 in NICU	Emergency caesarean delivery (vs. elective)	0.72	0.17–3.02	0.655
FiO_2_ > 0.21 in NICU	Antenatal corticosteroid administration	2.62	0.70–9.73	0.151

aOR, adjusted odds ratio; CI, confidence interval; PAS, placenta accreta spectrum; NICU, neonatal intensive care unit. Antenatal corticosteroid administration was coded as yes versus no.

## Data Availability

The dataset supporting this study has been deposited in a public repository and is accessible at the following URL: https://doi.org/10.17605/OSF.IO/C4XN2 (accessed on 22 March 2026).

## Supplementary Materials

The following supporting information can be downloaded at: https://www.mdpi.com/article/10.3390/jcm15155891/s1, File S1: STROBE Statement—Checklist of items that should be included in reports of cohort studies.

## Abbreviations

The following abbreviations are used in this manuscript:

PAS	placenta accreta spectrum
NICU	neonatal intensive care unit
STROBE	Strengthening the Reporting of Observational Studies in Epidemiology
RDS	respiratory distress syndrome
TTN	transient tachypnoea of the newborn
IVH	intraventricular haemorrhage
CPAP	continuous positive airway pressure
NIV	non-invasive ventilation
PEEP	positive end-expiratory pressure
PIP	peak inspiratory pressure
CRP	C-reactive protein
VEGF	vascular endothelial growth factor
IQR	interquartile range
OR	odds ratio
aOR	adjusted odds ratio
CI	confidence interval
EPV	events per variable
RR	risk ratio
FiO_2_	fraction of inspired oxygen
