# Supplementary material for: Neonatal Respiratory Outcomes in Placenta Previa with Histopathologically Confirmed Placenta Accreta Spectrum
Source: J Clin Med. 2026 Jul 28;15(15):5891. doi: 10.3390/jcm15155891 (PMC13466914; doi:10.3390/jcm15155891)
Supplement: Supplementary file 1 [file jcm-15-05891-s001.zip › jcm-4379949-supplementary.pdf]

Supplementary File S1

STROBE Statement—Checklist of items that should be included in reports of cohort studies

Manuscript: Neonatal Respiratory Outcomes in Placenta Previa with Histopathology-Confirmed Placenta Accreta Spectrum

Page numbers refer to the revised manuscript.

Section/topic	Item No.	Recommendation	Reported on page/section
**Title and abstract**	1(a)	Indicate the study's design with a commonly used term in the title or the abstract.	p. 1 (Abstract: retrospective single-centre cohort study)
**Title and abstract**	1(b)	Provide in the abstract an informative and balanced summary of what was done and what was found.	p. 1 (Abstract)
**Introduction**			
**Background/rationale**	2	Explain the scientific background and rationale for the investigation being reported.	pp. 2-3 (Introduction)
**Objectives**	3	State specific objectives, including any prespecified hypotheses.	p. 3 (final paragraph of Introduction)
**Methods**			
**Study design**	4	Present key elements of study design early in the paper.	p. 3 (Materials and Methods)
**Setting**	5	Describe the setting, locations, and relevant dates, including periods of recruitment, exposure, follow-up, and data collection.	pp. 3-4 (institution, study period 2014-2021, and perinatal management)
**Participants**	6(a)	Give the eligibility criteria, and the sources and methods of selection of participants. Describe methods of follow-up.	p. 3 (all consecutive patients; inclusion/exclusion criteria and histopathological classification)
**Participants**	6(b)	For matched studies, give matching criteria and number of exposed and unexposed.	Not applicable - the study was not matched.
**Variables**	7	Clearly define all outcomes, exposures, predictors, potential confounders, and effect modifiers. Give diagnostic criteria, if applicable.	pp. 3-5 (PAS exposure, respiratory and secondary outcomes, covariates, and diagnostic definitions)
**Data sources/measurement**	8*	For each variable of interest, give sources of data and details of methods of assessment (measurement). Describe comparability of assessment methods if there is more than one group.	pp. 3-5 (maternal, neonatal, and histopathological medical records; identical assessment framework in both groups)
**Bias**	9	Describe any efforts to address potential sources of bias.	pp. 3-6 (consecutive inclusion, histopathological confirmation, no imputation, prespecified parsimonious adjustment and sensitivity analyses); pp. 11-12 (limitations)
**Study size**	10	Explain how the study size was arrived at.	p. 6 (all eligible cases included; no prospective sample-size calculation because of retrospective design and rarity of PAS)
**Quantitative variables**	11	Explain how quantitative variables were handled in the analyses. If applicable, describe which groupings were chosen and why.	pp. 5-6 (medians with IQRs; categorical thresholds and continuous gestational age in regression)
**Statistical methods**	12(a)	Describe all statistical methods, including those used to control for confounding.	pp. 5-6 (comparative tests, odds ratios, logistic regression, covariate selection, and EPV considerations)
**Statistical methods**	12(b)	Describe any methods used to examine subgroups and interactions.	Not applicable - no subgroup or interaction analyses were performed.
**Statistical methods**	12(c)	Explain how missing data were addressed.	pp. 4-5 (available-case descriptive analyses, complete-case sensitivity analyses, and no imputation)
**Statistical methods**	12(d)	If applicable, explain how loss to follow-up was addressed.	Not applicable - retrospective record-based cohort without longitudinal follow-up.
**Statistical methods**	12(e)	Describe any sensitivity analyses.	pp. 5-6 and pp. 8-9; Table 3 (additional adjustment for urgency of caesarean delivery and antenatal corticosteroids)
**Results**			
**Participants**	13(a)*	Report numbers of individuals at each stage of study - e.g., numbers potentially eligible, examined for eligibility, confirmed eligible, included in the study, completing follow-up, and analysed.	pp. 3-4 and p. 6; Figure 1 (82 included: 37 PAS and 45 non-PAS; primary respiratory data available for 80)
**Participants**	13(b)*	Give reasons for non-participation at each stage.	p. 3 (cases without placental histopathology were excluded; exact initial screened and excluded numbers could not be reliably reconstructed)
**Participants**	13(c)*	Consider use of a flow diagram.	p. 4, Figure 1
**Descriptive data**	14(a)*	Give characteristics of study participants and information on exposures and potential confounders.	p. 6; Table 1
**Descriptive data**	14(b)*	Indicate number of participants with missing data for each variable of interest.	pp. 6-8; Tables 1 and 2 (n/N denominators and available observations)
**Descriptive data**	14(c)*	Summarise follow-up time (e.g., average and total amount).	Not applicable - no longitudinal follow-up period.

Section/topic	Item No.	Recommendation	Reported on page/section
**Outcome data**	15*	Report numbers of outcome events or summary measures over time.	pp. 7-8; Table 2
**Main results**	16(a)	Give unadjusted estimates and, if applicable, confounder-adjusted estimates and their precision (e.g., 95% confidence interval). Make clear which confounders were adjusted for and why they were included.	pp. 7-9; Tables 2 and 3; covariate rationale on pp. 5-6
**Main results**	16(b)	Report category boundaries when continuous variables were categorised.	pp. 4-5 and p. 8; Table 2 (Apgar, umbilical pH, and FiO2 thresholds)
**Main results**	16(c)	If relevant, consider translating estimates of relative risk into absolute risk for a meaningful time period.	Not applicable - outcomes were binary perinatal events rather than time-to-event risks.
**Other analyses**	17	Report other analyses done - e.g., analyses of subgroups and interactions, and sensitivity analyses.	pp. 8-9; Table 3 (exploratory sensitivity analyses)
**Discussion**			
**Key results**	18	Summarise key results with reference to study objectives.	pp. 10-12 (Discussion); p. 12 (Conclusions)
**Limitations**	19	Discuss limitations of the study, taking into account sources of potential bias or imprecision. Discuss both direction and magnitude of any potential bias.	pp. 11-12 (limitations paragraph)
**Interpretation**	20	Give a cautious overall interpretation of results considering objectives, limitations, multiplicity of analyses, results from similar studies, and other relevant evidence.	pp. 10-12 (Discussion and Conclusions)
**Generalisability**	21	Discuss the generalisability (external validity) of the study results.	pp. 11-12 (single-centre setting and need for larger multicentre cohorts)
**Other information**			
**Funding**	22	Give the source of funding and the role of the funders for the present study and, if applicable, for the original study on which the present article is based.	p. 12 (Funding source stated)

* Give information separately for exposed and unexposed groups where applicable. In this manuscript, these correspond to the PAS and non-PAS groups.

Abbreviations: PAS, placenta accreta spectrum; IQR, interquartile range; EPV, events per variable; FiO2, fraction of inspired oxygen.

Checklist completed using the STROBE cohort-study checklist.
